# Effects of Acute Ingestion of Caffeine Capsules on Muscle Strength and Muscle Endurance: A Systematic Review and Meta-Analysis

**DOI:** 10.3390/nu16081146

**Published:** 2024-04-12

**Authors:** Weiliang Wu, Zhizhou Chen, Huixuan Zhou, Leiyuyang Wang, Xiang Li, Yuanyuan Lv, Tingting Sun, Laikang Yu

**Affiliations:** 1 Key Laboratory of Physical Fitness and Exercise, Ministry of Education, Beijing Sport University, Beijing 100084, China; wwwuweiliang@163.com; 2 Department of Strength and Conditioning Assessment and Monitoring, Beijing Sport University, Beijing 100084, China; 730415czz@sina.com (Z.C.); ningyang191@outlook.com (L.W.); jinaxun0889@yeah.net (X.L.); 3 School of Sport Sciences, Beijing Sport University, Beijing 100084, China; chouhuixuan@live.cn; 4 China Institute of Sport and Health Science, Beijing Sport University, Beijing 100084, China; sunflowerlyy@bsu.edu.cn

**Keywords:** caffeine capsules, muscle strength, muscle endurance, systematic review, meta-analysis

## Abstract

This study aimed to explore the effects of acute ingestion of caffeine capsules on muscle strength and muscle endurance. We searched the PubMed, Web of Science, Cochrane, Scopus, and EBSCO databases. Data were pooled using the weighted mean difference (WMD) and 95% confidence interval. Fourteen studies fulfilled the inclusion criteria. The acute ingestion of caffeine capsules significantly improved muscle strength (WMD, 7.09, *p* < 0.00001) and muscle endurance (WMD, 1.37; *p* < 0.00001), especially in males (muscle strength, WMD, 7.59, *p* < 0.00001; muscle endurance, WMD, 1.40, *p* < 0.00001). Subgroup analyses showed that ≥ 6 mg/kg body weight of caffeine (WMD, 6.35, *p* < 0.00001) and ingesting caffeine 45 min pre-exercise (WMD, 8.61, *p* < 0.00001) were more effective in improving muscle strength, with the acute ingestion of caffeine capsules having a greater effect on lower body muscle strength (WMD, 10.19, *p* < 0.00001). In addition, the acute ingestion of caffeine capsules had a greater effect in moderate-intensity muscle endurance tests (WMD, 1.76, *p* < 0.00001). An acute ingestion of caffeine capsules significantly improved muscle strength and muscle endurance in the upper body and lower body of males.

## 1. Introduction

Caffeine is widely available in a variety of beverages and nutritional supplements as a central nervous stimulant with energizing effects. Since it was delisted by the World Anti-Doping Agency (WADA) in 2004, elite athletes have been extensively utilizing caffeine as a tool to boost their training and competitive performance [1]. A previous study showed that around 73.8% of athletes ingest caffeine prior to or during competition, with a higher percentage of endurance athletes [2]. As of 2015, caffeine is used by up to 76% of elite athletes, with the most widespread use among athletes in track and field, cycling, and rowing [3]. The potentiating effect of caffeine on sports performance is well recognized. Typically, athletes consume a dose of 3–6 mg/kg body weight (BW) caffeine in pill or capsule form 30–90 min before exercise [4].

Muscle strength and endurance, the foundational abilities of athletes, are closely related to sports performance. Previous studies have shown that even low doses of caffeine ingestion can significantly enhance muscle strength [5], regardless of habitual caffeine consumption [6]. A previous study reported that acute ingestion of caffeine leads to a significant enhancement in isokinetic strength [7]. However, Wilk et al. [8] showed that high acute caffeine ingestion did not result in any improvement in muscle strength, which may be due to the fact that caffeine tolerance varies in different individuals, and that high caffeine ingestion doses can lead to side effects, which may affect the potentiating effect.

Furthermore, there is ongoing debate surrounding the impact of acute caffeine ingestion on muscle endurance. Duncan et al. [9] demonstrated a marked rise in the number of repetitions performed, coupled with a substantial enhancement in both upper and lower body muscle endurance, subsequent to acute caffeine ingestion. Southward et al. [10] further verified the notable impact of caffeine ingestion on muscle endurance. This effect can be attributed to the mechanism where caffeine triggers the release of norepinephrine, acetylcholine, dopamine, and serotonin by binding to adenosine receptors A1 and A2A [11]. This, in turn, enhances muscle tone [12] and diminishes the suppressive influence of adenosine on neurotransmission, excitation, and nociception [13]. In addition, caffeine delays fatigue by blocking the negative effects of adenosine receptors on the central nervous system [14].

However, Beck et al. [15] observed no notable effect of consuming caffeine-containing supplements on upper body muscle endurance, which aligned with the results of Grgic et al. [16]. Their study revealed that caffeine ingestion failed to augment the number of repetitions during resistance training. Collectively, these findings imply that caffeine ingestion does not significantly boost muscle endurance. This variation may stem from the distinct muscle composition of the upper and lower body and the large differences in activated and mobilized muscle fibers, which affect the potentiation of caffeine [17]. Furthermore, different genotypes of individuals have different sensitivities to caffeine [18], which is also an important factor to consider.

A prior study showed that caffeine ingestion led to an enhancement in both muscle strength and muscle endurance, but was limited to the muscle performance of knee extensors [19]. Moreover, Grgic et al. [20] exhibited that caffeine ingestion significantly bolstered muscle strength and endurance, albeit exclusively in upper body muscle groups, with the subjects solely comprising females. Additionally, Southward et al. [10] revealed that caffeine ingestion notably enhanced muscle endurance, but specifically in the context of cycling tests.

Caffeine is primarily administered in the form of capsules, and its efficacy in enhancing muscle strength and endurance is mostly established with this form. Therefore, this study aimed to explore the effects of an acute ingestion of caffeine capsules on muscle strength and endurance.

## 2. Materials and Methods

### 2.1. Design

This study was conducted in accordance with the criteria and recommendations of the Preferred Reporting Items for Systematic Evaluation and Meta-Analysis [21]. The protocol was registered with PROSPERO under the registration number CRD42023424824.

### 2.2. Search Strategy

We searched the PubMed, EBSCO, Cochrane, Web of science, and Scopus databases from the inception dates to 19 May 2023, using the following keywords and MESH terms: caffeine, muscle strength, and muscle endurance. We also manually searched references listed in the identified systematic reviews and meta-analyses. Two authors (W.W. and Z.C.) independently completed the article screening using a standardized form.

### 2.3. Eligibility Criteria

Inclusion criteria were as follows: (1) RCTs; (2) including acute caffeine ingestion group and placebo ingestion group; (3) caffeine was provided using capsules; and (4) acute rather than long-term interventions. Exclusion criteria were as follows: (1) publications that were not in English; (2) conference papers; (3) review articles; and (4) studies conducted on animals.

### 2.4. Data Extraction

WW and ZC independently conducted the data extraction process, primarily focusing on the following aspects: (1) study characteristics (first author’s surname, publication year); (2) subject characteristics (age, gender, *n*, training experience); (3) intervention characteristics (dosage and timing of caffeine ingestion); and (4) outcome characteristics (muscle group location, intensity of muscle endurance tests).

### 2.5. Outcomes

The primary outcomes were muscle strength and muscle endurance. Muscle strength was measured using the maximum load lifted in a single effort, whereas muscle endurance was measured using the maximum number of repetitions achieved at a fixed load.

### 2.6. Methodological Quality Assessment

The methodological quality of the included studies was evaluated using the Cochrane risk of bias tool [22,23]. In addition, we also used the Physiotherapy Evidence Database (PEDro) scale to further assess the quality of the included studies. For PEDro scale, 11 items were evaluated, where studies scoring <4 points, 4–5 points, 6–8 points, and >9 points are considered poor, average, good, and excellent quality, respectively [24].

### 2.7. Statistical Analysis

From each study, we extracted mean and standard deviation (SD) values pertaining to muscle strength and muscle endurance in both the caffeine and placebo ingestion groups. To estimate the impact of acute caffeine ingestion on these parameters, we employed the weighted mean difference (WMD) along with a 95% confidence interval (CI). For studies that reported standard errors (SE) and 95% CIs, we calculated the SD according to previous studies [19,20]. In cases where high heterogeneity was observed, we utilized subgroup analysis, meta-regression analysis, and sensitivity analysis to interpret the results [25,26].

For subgroup analyses, we tried to investigate the impact of acute ingestion of caffeine capsules on muscle strength using muscle group location (upper or lower body), dose of caffeine ingestion (<6 or ≥6 mg/kg BW), timing of caffeine ingestion (45 min or 60 min pre-exercise), and participant gender (male or female). Similarly, we explored the effects of acute caffeine ingestion on muscle endurance using muscle group location (upper or lower body), intensity of the muscle endurance tests (low-, moderate-, or high-intensity), and participant gender (male or female). The forest plots were generated using RevMan software (Version 5.4), and sensitivity analysis, funnel plot, and meta-regression were performed using Stata software (Version 15.0). Statistical significance was considered for outcomes with a *p* < 0.05.

## 3. Results

### 3.1. Studies Selection

**Figure 1:**
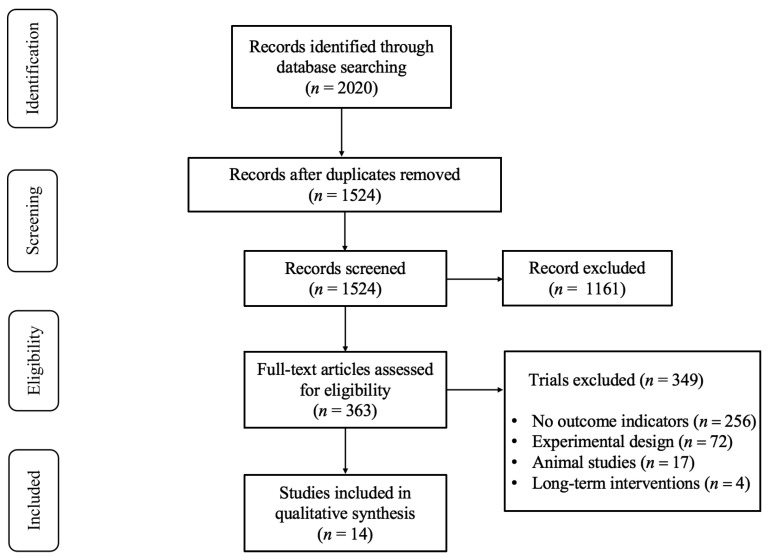
PRISMA Flow Diagram of the Study Selection Process.

Figure 1 illustrates the initial retrieval of 2020 records from the databases. A total of 1524 studies remained after excluding duplicates and 363 potentially eligible studies remained after the title and abstract screen. Upon reading the full text, 349 studies were excluded for the following reasons: (1) they investigated irrelevant outcomes (*n* = 256); (2) the caffeine ingestion group was combined with other interventions (*n* = 72); (3) they were animal studies (*n* = 17); and (4) they reported long-term interventions (*n* = 4). Finally, 14 studies [6,11,27,28,29,30,31,32,33,34,35,36,37,38] met the inclusion criteria.

### 3.2. Characteristics of the Included Studies

Table 1 presents the characteristics of caffeine ingestion and participants. Of the 14 studies, 11 studies involved only males [11,27,28,29,31,32,33,34,35,37,38] and 3 studies involved only females [6,30,36]. Eight studies provided data for muscle strength [6,27,28,30,33,34,36,38], which was tested using one repetition maximum (1 RM). In addition, 13 studies provided data for muscle endurance [6,11,28,29,30,31,32,33,34,35,36,37,38], which was measured using the maximum number of repetitions achieved at a fixed load. The dose of caffeine ingestion ranged from 2 to 11 mg/kg BW, and the timing of caffeine ingestion was 45 min or 60 min. In terms of the intensity of muscle endurance tests, three studies involved low-intensity muscle endurance tests (<60% 1 RM) [6,30,34], seven studies involved moderate-intensity muscle endurance tests (60−85% 1 RM) [11,28,31,33,35,36,38], and three studies involved high-intensity muscle endurance tests (≥85% 1 RM) [29,31,32].

### 3.3. Meta-Analysis

**Figure 2:**
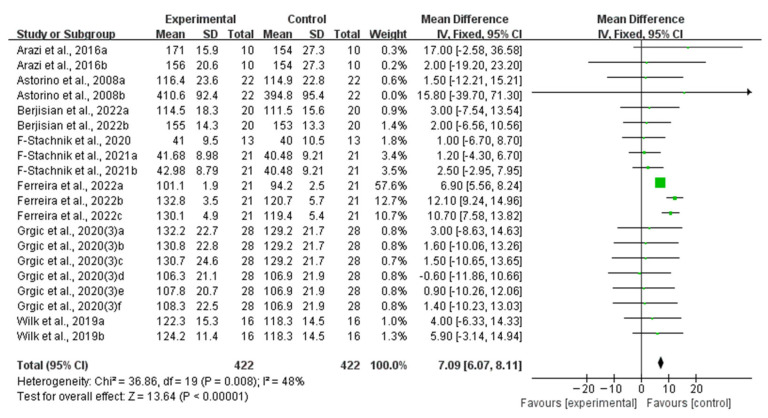
Meta-analysis results of the effects of acute ingestion of caffeine capsules on muscle strength [6,27,28,30,33,34,36,38]. Diamonds indicated the effect size of each study summarized as WMD. The size of the shaded squares was proportional to the percentage weight of each study. Horizontal lines represented the 95% CI and the vertical line represented the overall effect.

**Figure 3:**
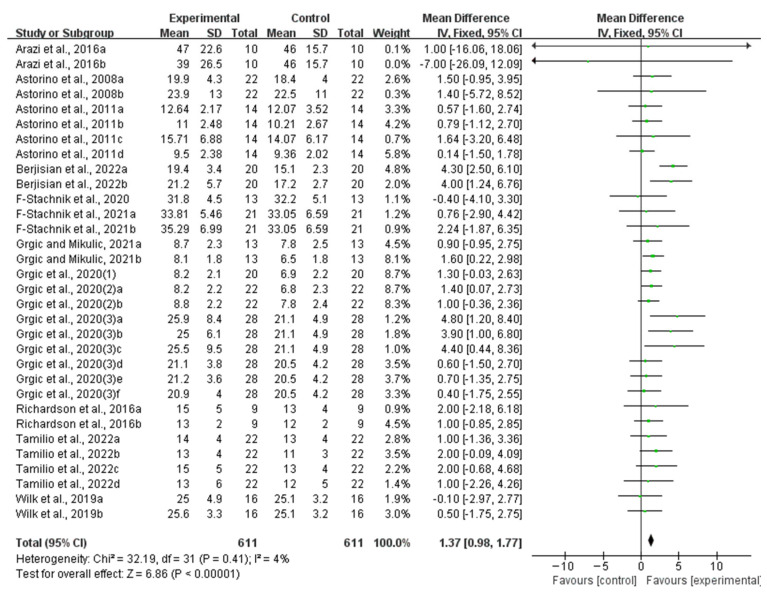
Meta-analysis results of the effect of acute ingestion of caffeine capsules on muscle endurance [6,11,28,29,30,31,32,33,34,35,36,37,38]. Diamonds indicated the effect size of each study summarized as WMD. The size of the shaded squares was proportional to the percentage weight of each study. Horizontal lines represented the 95% CI and the vertical line represented the overall effect.

Compared with the placebo ingestion group, an acute ingestion of caffeine capsules significantly improved muscle strength (WMD, 7.09, 95% CI, 6.07 to 8.11, *p* < 0.00001, *I*^2^ = 48%, Figure 2) and muscle endurance (WMD, 1.37, 95% CI, 0.98 to 1.77, *p* < 0.00001, *I*^2^ = 4%, Figure 3).

### 3.4. Meta-Regression Analysis

Meta-regression analyses were conducted to explore the effects of the dose and the timing of caffeine ingestion. There was a significant association between the dose (*p* = 0.021) or the timing of caffeine ingestion (*p* = 0.003) and muscle strength (Figure S1). However, no significant associations were observed between the timing of caffeine ingestion (*p* = 0.966) or the dose of caffeine ingestion (*p* = 0.571) and muscle endurance (Figure S2).

### 3.5. Subgroup Analysis

#### 3.5.1. Muscle Strength

**Figure 4:**
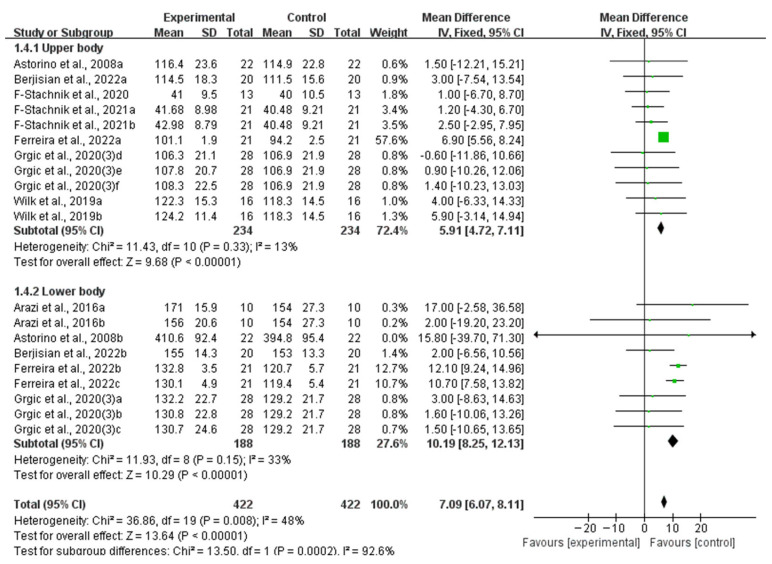
Meta-analysis results of the effects of acute ingestion of caffeine capsules on muscle strength in upper body and lower body [6,27,28,30,33,34,36,38]. Diamonds indicated the effect size of each study summarized as WMD. The size of the shaded squares was proportional to the percentage weight of each study. Horizontal lines represented the 95% CI and the vertical line represented the overall effect.

Stratifying the analysis by muscle group location, improvements in muscle strength remained significant in the upper body (WMD, 5.91, 95% CI, 4.72 to 7.11, *p* < 0.00001, *I*^2^ = 13%) and the lower body (WMD, 10.19, 95% CI, 8.25 to 12.13, *p* < 0.00001, *I*^2^ = 33%, Figure 4), while an acute ingestion of caffeine capsules exhibited a more pronounced effect on lower body muscle strength.

**Figure 5:**
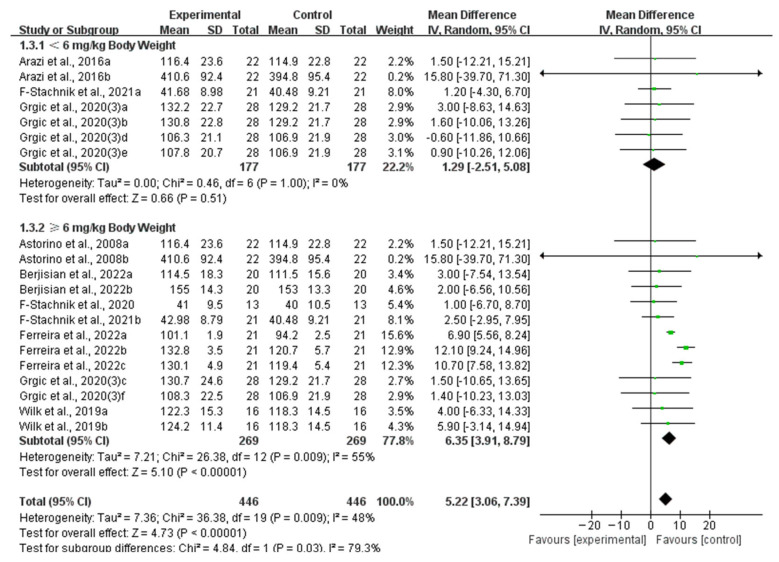
Meta-analysis results of the effects of dose of caffeine ingestion on muscle strength [6,27,28,30,33,34,36,38]. Diamonds indicated the effect size of each study summarized as WMD. The size of the shaded squares was proportional to the percentage weight of each study. Horizontal lines represented the 95% CI and the vertical line represented the overall effect.

Seven studies provided data for 6 mg/kg BW of caffeine ingestion, five studies provided data for 3 mg/kg BW of caffeine ingestion, two studies provided data for 2 mg/kg BW of caffeine ingestion, two studies provided data for 5 mg/kg BW of caffeine ingestion, and one study provided data for 4, 8, 9, and 11 mg/kg BW of caffeine ingestion. When analyzing the subgroup by dose of caffeine ingestion, ≥6 mg/kg BW of caffeine significantly improved muscle strength (WMD, 6.35, 95% CI, 3.91 to 8.79, *p* < 0.00001, *I*^2^ = 55%), while <6 mg/kg BW of caffeine did not significantly improve muscle strength (WMD, 1.29, 95% CI, −2.51 to 5.08, *p* = 0.51, *I*^2^ = 0%, Figure 5).

**Figure 6:**
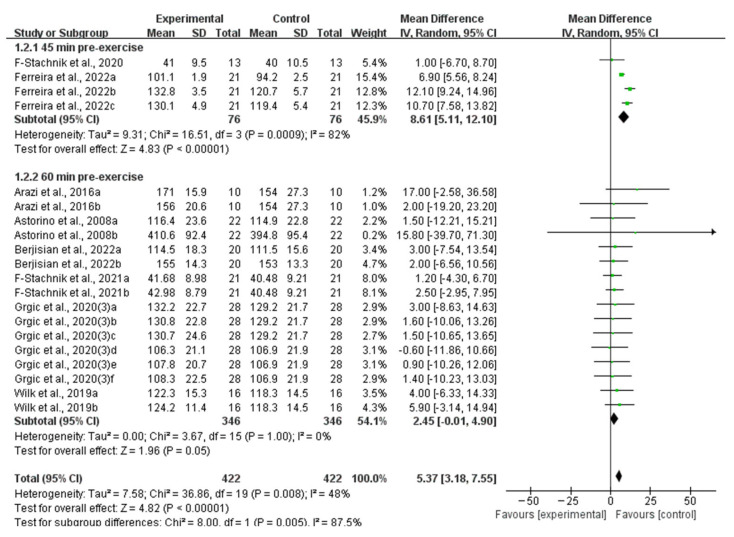
Meta-analysis results of the effects of timing of caffeine ingestion on muscle strength [6,27,28,30,33,34,36,38]. Diamonds indicated the effect size of each study summarized as WMD. The size of the shaded squares was proportional to the percentage weight of each study. Horizontal lines represented the 95% CI and the vertical line represented the overall effect.

Eleven studies provided data for ingesting caffeine 60 min pre-exercise and three studies provided data for ingesting caffeine 45 min pre-exercise. When analyzing the subgroup by timing of caffeine ingestion, ingesting caffeine 45 min pre-exercise significantly improved muscle strength (WMD, 8.61, 95% CI, 5.11 to 12.10, *p* < 0.00001, *I*^2^ = 82%), while ingesting caffeine 60 min pre-exercise showed no significant improvement in muscle strength (WMD, 2.45, 95% CI, −0.01 to 4.90, *p* = 0.05, *I*^2^ = 0%, Figure 6).

**Figure 7:**
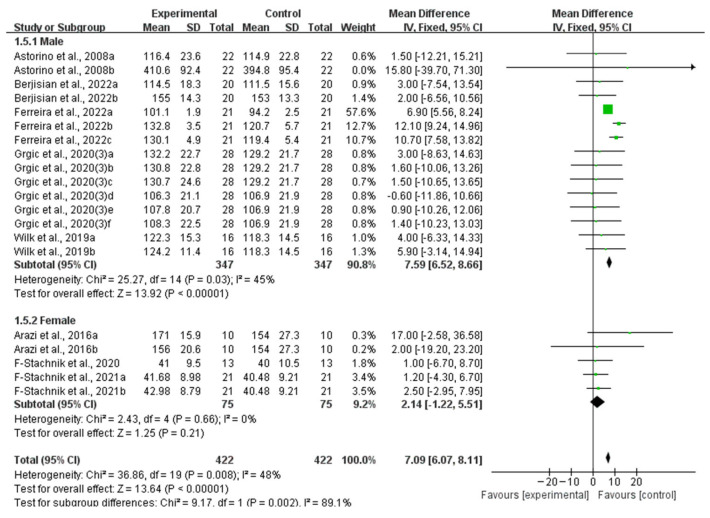
Meta-analysis results of the effects of acute ingestion of caffeine capsules on muscle strength in males and females [6,27,28,30,33,34,36,38]. Diamonds indicated the effect size of each study summarized as WMD. The size of the shaded squares was proportional to the percentage weight of each study. Horizontal lines represented the 95% CI and the vertical line represented the overall effect.

Five studies provided data for male participants and three studies provided data for female participants. When analyzing the subgroup by participant gender, only the subgroup of studies involving males exhibited significantly improved muscle strength (WMD, 7.59, 95% CI, 6.52 to 8.66, *p* < 0.00001, *I*^2^ = 45%), while the subgroup involving females did not exhibit significantly improved muscle strength (WMD, 2.14, 95% CI, −1.22 to 5.51, *p* = 0.21, *I*^2^ = 0%, Figure 7).

#### 3.5.2. Muscle Endurance

**Figure 8:**
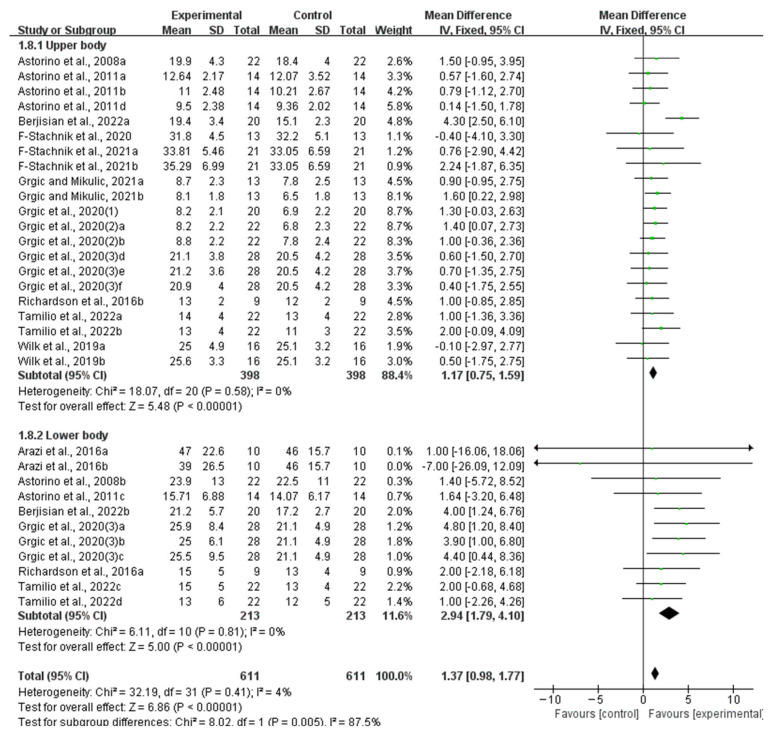
Meta-analysis results of the effect of acute ingestion of caffeine capsules on muscle endurance in upper body and lower body [6,11,28,29,30,31,32,33,34,35,36,37,38]. Diamonds indicated the effect size of each study summarized as WMD. The size of the shaded squares was proportional to the percentage weight of each study. Horizontal lines represented the 95% CI and the vertical line represented the overall effect.

Stratifying the analysis by muscle group location, the improvement in muscle endurance remained significant in the upper body (WMD, 1.17, 95% CI, 0.75 to 1.59, *p* < 0.00001, *I*^2^ = 0%) and the lower body (WMD, 2.94, 95% CI, 1.79 to 4.10, *p* < 0.00001, *I*^2^ = 0%, Figure 8), while an acute ingestion of caffeine capsules exhibited a more pronounced effect on lower body muscle endurance.

**Figure 9:**
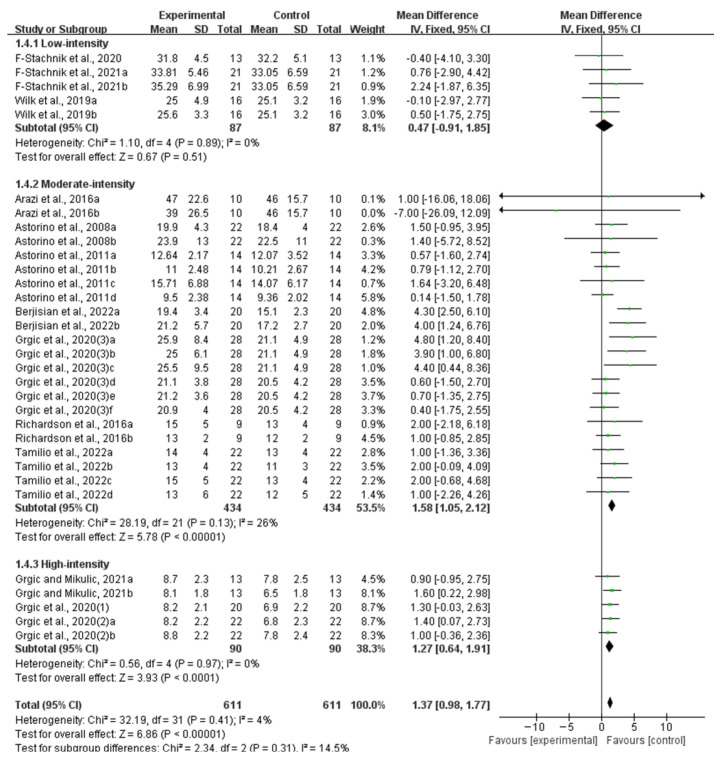
Meta-analysis results of the effect of acute ingestion of caffeine capsules on low-intensity, moderate-intensity, and high-intensity muscle endurance tests [6,11,28,29,30,31,32,33,34,35,36,37,38]. Diamonds indicated the effect size of each study summarized as WMD. The size of the shaded squares was proportional to the percentage weight of each study. Horizontal lines represented the 95% CI and the vertical line represented the overall effect.

Acute caffeine ingestion showed a significant improvement in the moderate-intensity (WMD, 1.58, 95% CI, 1.05 to 2.12, *p* < 0.00001, *I*^2^ = 26%) and the high-intensity muscle endurance tests (WMD, 1.27, 95% CI, 0.98 to 1.77, *p* < 0.00001, *I*^2^ = 4%). However, acute caffeine ingestion showed no significant improvement in the low-intensity muscle endurance tests (WMD, 0.47, 95% CI, −0.91 to 1.85, *p* = 0.51, *I*^2^ = 0%, Figure 9). The subgroup analysis indicated that an acute ingestion of caffeine capsules exhibited a more pronounced effect on the moderate-intensity muscle endurance tests.

**Figure 10:**
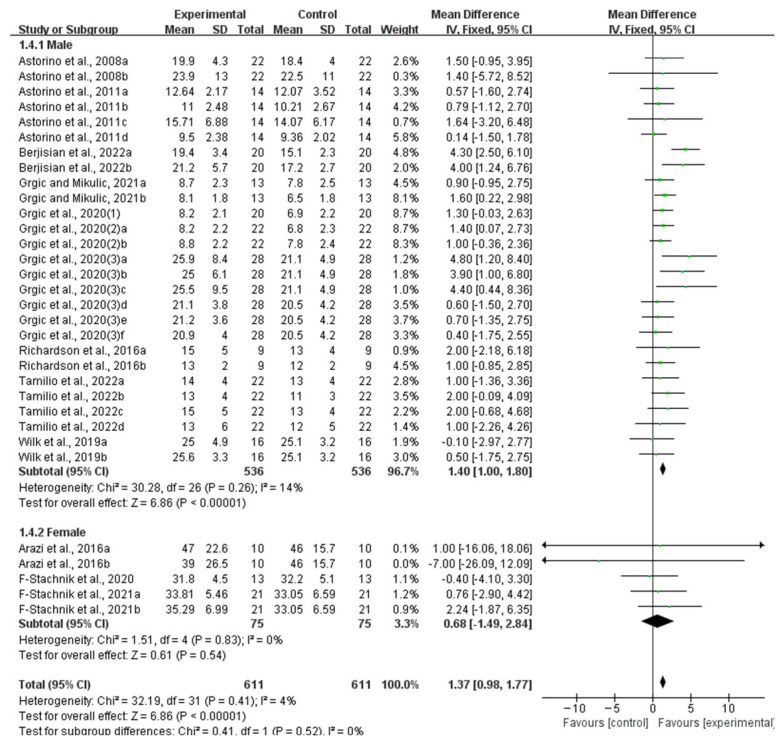
Meta-analysis results of the effect of acute ingestion of caffeine capsules on muscle endurance in males and females [6,11,28,29,30,31,32,33,34,35,36,37,38]. Diamonds indicated the effect size of each study summarized as WMD. The size of the shaded squares was proportional to the percentage weight of each study. Horizontal lines represented the 95% CI and the vertical line represented the overall effect.

In the subgroup analysis by participant gender, only the subgroup of studies involving males showed a significant improvement in muscle endurance (WMD, 1.40, 95% CI, 1.00 to 1.80, *p* < 0.00001, *I*^2^ = 14%), while the subgroup involving females showed no significant improvement in muscle endurance (WMD, 0.68, 95% CI, −1.49 to 2.84, *p* = 0.54, *I*^2^ = 0%, Figure 10).

### 3.6. Risk of Bias

We used the Cochrane risk of bias tool to assess the quality of the included studies in terms of biases such as selection, performance, detection, attrition, reporting, and others (Figure S3). Using the PEDro scale, it was determined that out of the 14 included studies, 3 were rated as excellent in quality, and 11 were classified as good quality (Table 2).

### 3.7. Publication Bias

Possible publication bias was assessed using a funnel plot (Figure S4). A visual inspection of the funnel plot suggests the absence of funnel plot asymmetry. Based on the results of the Egger’s test, small-sample-size studies were insufficient to affect the final results (muscle strength, *p* = 0.055, Table 3; muscle endurance, *p* = 0.577, Table 4); we also performed the Dsuval and Tweedie’s trim and fill procedure, and the results indicated that the combined results were robust.

### 3.8. Sensitivity Analysis

Sensitivity analyses revealed that the overall effect of acute caffeine ingestion on muscle strength (Figure S5) and muscle endurance (Figure S6) remained consistent in terms of direction and compatibility levels when any of the included studies were omitted.

## 4. Discussion

The objective of this study was to investigate the effects of an acute ingestion of caffeine capsules on muscle strength and endurance. Fourteen studies were included, and the findings indicated that an acute ingestion of caffeine capsules significantly improved upper and lower body muscle strength and endurance among male individuals. Subgroup analyses showed that a caffeine ingestion of ≥6 mg/kg BW and 45 min prior to exercise was more effective in improving muscle strength. Additionally, acute caffeine ingestion led to notable improvements in moderate- and high-intensity muscle endurance tests, with a more pronounced effect observed in moderate-intensity endurance tests.

### 4.1. Effects of Caffeine on Muscle Strength

The ingestion of caffeine is likely to exert an ergogenic effect on 1 RM strength [39,40]. Our results showed that an acute ingestion of caffeine capsules significantly improved muscle strength, which was in agreement with the results of previous studies [41,42]. Acute caffeine ingestion led to a significant enhancement of muscle strength in professional strength trainers [8], amateur trainers [43], team sport athletes [44], individual competitive athletes [45], males [46], and females [47]. The main mechanism by which caffeine enhances muscle strength is that caffeine stimulates the central nervous system and improves neuromuscular transmission by facilitating Ca^2+^ release from the sarcoplasmic reticulum [34], and inhibits Ca^2+^ reuptake to increase muscle contractility, ultimately leading to an increase in muscle strength [48]. In addition, it also increases the recruitment of motor units and inhibits Ca^2+^ reabsorption [49].

In addition, when caffeine is ingested, it promptly appears in the bloodstream, with blood levels escalating between 15 and 45 min after consumption. Previous studies have demonstrated that peak plasma concentrations of caffeine typically arise 30 to 120 min following oral administration [4,50]. After being rapidly and completely absorbed by the gastrointestinal tract, caffeine is efficiently distributed throughout all tissues of the body. Some studies indicate that the average distribution half-life of caffeine following ingestion is 0.2 h, while the elimination half-life ranges from 2.5 to 10 h, averaging 3.8 h [51,52]. Although the typical half-life of caffeine commonly falls between 4 and 6 h, there are individual variations, and the half-life in adults can even span from 1.5 to 10 h [53].

On the contrary, Collier et al. [54] explored the effect of caffeine-containing energy drinks on muscle strength. The study used Full Throttle Original Citrus, which contained carbonated water, caffeine (100 mg per 237 mL serving), high-fructose corn syrup, sucrose (carbohydrate totaling 29 g per 237 mL serving), taurine (amount not disclosed), natural and artificial flavors, citric acid, sodium benzoate, ginseng extract, guarana extract, acacia, carnitine fumarate, vitamin B3, vitamin B6, vitamin B12, glycerol ester of wood rosin, and yellow 5. The results showed that acute caffeine ingestion had little effect on muscle strength, which might be due to the fact that the ingestion of caffeine-containing energy drinks includes other substances that could affect the results.

#### 4.1.1. Muscle Group Location

A previous meta-analysis has shown that the effects of caffeine on muscle strength are affected by muscle group size [41]. Therefore, we performed a subgroup analysis based on the location of muscle groups and showed that an acute ingestion of caffeine capsules significantly improved upper body and lower body muscle strength, whereas acute caffeine ingestion exhibited a more pronounced effect on lower body muscle strength, which was consistent with a previous meta-analysis [19], showing that the effect of caffeine is linked to the size and position of the muscle group, which correlates with an elevation in the number of adenosine receptors. That is, the larger the muscle mass, the better potentiating effect of caffeine. In addition, this is also supported by a randomized, crossover, double-blind study, which reported that among young men who underwent resistance training, caffeine ingestion significantly improved the mean isometric peak torque of larger muscle groups compared to smaller muscle groups [55]. Furthermore, a randomized, subject-blinded crossover study showed that the overall effect of caffeine on the lower body muscle groups of resistance-trained men was 4–6 times greater than that on the upper body [56]. This may be due to the larger muscle groups in the lower body with a higher number of activated and mobilized muscle fibers, which increases motor unit recruitment and muscle fiber conduction velocity [17]. Moreover, larger muscle groups have lower neural activation, and the potentiation is largely attributed to the mechanistic action of caffeine in promoting motor unit recruitment through the central nervous system [57], which suggests that caffeine improves strength by activating the central nervous system, resulting in a more pronounced increase in motor unit recruitment in large muscle groups than in small muscle groups [58].

#### 4.1.2. Caffeine Dose

Grgic et al. [40] showed that ingesting lower dosages of caffeine exhibits comparable ergogenic effects to ingesting higher dosages of caffeine. However, our subgroup analysis showed a correlation between the dose of caffeine ingestion and muscle strength, with ≥6 mg/kg BW of caffeine significantly improving muscle strength, while <6 mg/kg BW of caffeine did not improve muscle strength, which was in agreement with a double-blind, randomized crossover study [36], which showed that an acute ingestion of 2 mg/kg BW of caffeine did not enhance muscle strength in female teenage karate athletes. A randomized, double-blind crossover study of upper body muscle strength in female habitual caffeine users showed that an acute ingestion of 6 mg/kg BW of caffeine resulted in a significant boost in barbell speed during the bench press, as compared to the ingestion of 3 mg/kg BW [59]. In addition, Nemati et al. [60] showed that 6 mg/kg BW of caffeine was more effective in enhancing lower body muscle strength in collegiate male volleyball players compared to 3 mg/kg BW. This may be due to the fact that caffeine at ≥6 mg/kg BW better promotes Ca^2+^ release and enhances the contractility of skeletal muscle, making the potentiating effect significant. Furthermore, the effect of caffeine on muscle is manifested by its ability to stimulate the metabolic process of muscle contraction [50], thereby increasing the contractile force, and ≥6 mg/kg BW of caffeine results in a faster metabolism of muscle contraction. Moreover, most of subjects included in the meta-analysis were non-habitual caffeine users with a high level of sensitivity to caffeine, and it is now generally accepted that 6 mg/kg BW is the intermediate dose, which is better potentiated in the majority of non-habitual caffeine users [38].

#### 4.1.3. Timing of Caffeine Ingestion

Our subgroup analysis showed that ingesting caffeine 45 min pre-exercise showed a significant improvement in muscle strength, while ingesting caffeine 60 min pre-exercise showed no significant improvement in muscle strength. This finding aligned with previous randomized, double-blind crossover studies [30,59], which demonstrated that among resistance-trained women, bench press performance significantly improved when 6 mg/kg BW of caffeine was ingested 45 min prior to exercise. However, in resistance-trained men, no significant difference was observed when caffeine was consumed 60 min before exercise. Grgic et al. [40] demonstrated that the optimal time for ingesting caffeine in capsule form is approximately 30 to 60 min prior to exercise, which was in line with the results of Harty et al. [61]. In that study, participants ingested 6 mg/kg BW of caffeine capsules 30 min, 60 min, or 120 min prior to exercise, and only caffeine ingested at 30 min significantly improved the absolute isokinetic knee extensor performance. In the studies we included, caffeine capsules were ingested either 45 or 60 min before exercise. Therefore, the optimal time interval for ingesting caffeine capsules to enhance muscle strength may be 45 min prior to exercise.

#### 4.1.4. Participant Gender

The participant’s gender could potentially influence the effect of acute caffeine ingestion on muscle strength. Our subgroup analysis revealed that only studies involving male participants demonstrated significant improvements in muscle strength, while the subgroup involving females showed no significant improvement in muscle strength. This finding aligned with a randomized, double-blind crossover study [61], which showed that an acute ingestion of caffeine capsules significantly improved the isometric peak torque in male participants and had no effect on female participants. In addition, a systematic review showed that male athletes exhibited greater improvements in total weight lifted compared to female athletes, despite receiving the same dose of caffeine [62]. The potential differences in gender response to caffeine may be attributed to the influence of sex hormones on gastrointestinal emptying. Multiple studies have demonstrated that elevated progesterone levels lead to slower gastric emptying, leading to a diminished potentiation following caffeine ingestion [63,64]. Furthermore, female participants may be in various phases of the menstrual cycle, resulting in fluctuating hormonal responses that can alter the metabolic rate of caffeine, which in turn affects the effects of caffeine [65]. In addition, previous studies have confirmed that trained athletes are more likely to achieve better strength performance after caffeine ingestion [4,66]. In the studies we included, males generally had more years of training than females, suggesting that training experience could be a contributing factor to the lack of muscle strength gains observed among female participants. However, it is also conceivable that physiological differences, such as testosterone levels or muscle fiber type, could play a role. Therefore, further prospective and interventional studies are needed.

### 4.2. Effects of Caffeine on Muscle Endurance

Our findings demonstrated that an acute ingestion of caffeine capsules significantly improved muscle endurance. This aligned with prior studies [67,68], which showed an increase in lower body muscle endurance by 8.8% and 11−12%, respectively, following acute caffeine ingestion. Furthermore, two meta-analyses revealed a 6–7% enhancement in muscle endurance compared to the placebo group [69], indicating the potentiation effect of acute caffeine ingestion on muscle endurance, which concurred with Lopes et al. [70]. Prior studies have also demonstrated that acute caffeine ingestion significantly augmented the mean power output of the knee extensors [55], thereby enhancing upper body muscle endurance in females [20]. The primary mechanism underlying the enhancement of muscle endurance through caffeine ingestion lies in its antagonism of adenosine receptors and its effects within the central nervous system [67]. During moderate- to high-intensity exercise, continuous muscle contractions can lead to minor damage to contractile proteins and trigger inflammation, resulting in pain. Caffeine ingestion helps alleviate this muscle discomfort and pain by increasing the concentration of plasma β-endorphins [71], thereby improving muscle endurance. Additionally, caffeine crosses the blood–brain barrier and binds to adenosine receptors, reducing the inhibitory effect of adenosine and subsequently enhancing muscle endurance [72].

However, a randomized, double-blind, placebo-controlled crossover study showed that 9 and 11 mg/kg BW of caffeine did not significantly enhance muscle endurance [8], which was contrary to the findings of this study. In an experiment conducted by Williams et al. [73], similar findings were noted, revealing that caffeine had no effect on muscle endurance. This may be due to the habitual ingestion of caffeine by the subjects, which resulted in reduced sensitivity after acute caffeine ingestion, thus weakening the potentiating effect of caffeine [8].

#### 4.2.1. Muscle Group Location

The size of muscle groups can influence the ergogenic impact of acute caffeine ingestion on muscle endurance [58]. Therefore, we performed a subgroup analysis based on the location of muscle groups and showed that an acute ingestion of caffeine capsules significantly improved upper body and lower body muscle endurance. Notably, the effect of an acute caffeine ingestion was more pronounced in enhancing lower body muscle endurance, aligning with the observations made by Ruiz-Fernández et al. [58]. This suggests that acute caffeine ingestion is more effective in improving muscle endurance in the lower body compared to the upper body. Furthermore, acute caffeine ingestion delayed the onset of fatigue, leading to an increase in the number of repetitions during upper body and lower body exercises. The enhancement in muscle endurance was more evident when considering muscle size groups. The different responses of the upper body and the lower body can be partly attributed to the fact that the number of adenosine receptors in muscle groups varies depending on their size [56]. 

#### 4.2.2. Intensity of Muscle Endurance Tests

The impact of acute caffeine ingestion on muscular endurance varies across different load ranges [65,74]. We observed a significant enhancement in moderate- and high-intensity muscle endurance tests following acute caffeine ingestion, while acute caffeine ingestion had no significant improvement in low-intensity muscle endurance tests. In addition, an acute ingestion of caffeine capsules had a greater effect in moderate-intensity muscle endurance tests, echoing findings from a double-blind, placebo-controlled crossover study [58]. This study revealed that acute caffeine ingestion significantly improved muscle endurance, particularly at loads of ≥75% 1 RM in resistance-trained athletes. This enhancement may be attributed to caffeine’s ability to block the parasympathetic effects of adenosine, reduce fatigue and perceived effort, and promote the replenishment of muscle fibers during muscle contraction [4]. However, when comparing moderate- and high-intensity exercises with low-intensity exercises, we hypothesize that low-intensity exercise may reduce the recruitment of motor units and that the inhibition of Ca^2+^ reuptake by the sarcoplasmic reticulum after caffeine ingestion may be diminished, failing to prolong the duration of muscle contractile activity. However, higher intensity loads may counteract the reduction in fatigue after caffeine ingestion, resulting in a loss of potentiating effect.

#### 4.2.3. Participant Gender

It is reported that participant gender could influence the effect of acute caffeine ingestion on muscle endurance. Males tend to respond more consistently to caffeine, displaying an ergogenic effect across multiple outcomes, in contrast to females [40]. Our results revealed a significant improvement in muscle endurance specifically among male-focused studies, whereas studies involving females did not show any significant enhancement. This aligned with the findings of Goldstein et al. [75], who observed no impact of acute caffeine ingestion on the number of bench press repetitions among resistance-trained females, which may be due to a weaker metabolic response to caffeine in females [76]. In addition, Wilk et al. [8] showed that there was no increase in muscle endurance after caffeine ingestion, possibly due to the adverse effects of higher caffeine doses, such as increased urinary output, tachycardia, gastrointestinal issues, and other side effects, which may interfere with the desired potentiating effect. Furthermore, a prior study exploring the effects of caffeine on muscle endurance and gender differences observed a trend towards improvement primarily among males, indicating a sex-specific ergogenic effect on muscle endurance [77]. This may be explained by the varying sensitivity to caffeine among different genders.

### 4.3. Limitations

This study is at risk of bias in terms of measurement bias, as only one study reported conducting the entire experiment for the same test persons, and none of the others reported blinded evaluations of the study outcomes. Additionally, there was a significant variance in the habitual caffeine ingestion among the subjects, which can influence the ergogenic effect of caffeine on muscle strength and endurance. Future studies may focus on habitual or non-habitual caffeine users.

## 5. Conclusions

Acute ingestion of caffeine capsules significantly improved the upper and lower body muscle strength and endurance among male individuals. In terms of muscle strength, a caffeine ingestion of ≥6 mg/kg BW and 45 min prior to exercise was more effective in improving muscle strength. In addition, acute ingestion of caffeine capsules significantly improved moderate- and high-intensity muscle endurance tests’ performance, with acute ingestion of caffeine capsules having a greater effect on moderate-intensity muscle endurance tests.

## Figures and Tables

**Table 1:** Characteristics of the studies included in this meta-analysis.

Study	Sample Size	Age (y)	Caffeine Dose (mg/kg BW)	Timing of Caffeine Ingestion (min)	Intensity of Muscle Endurance Tests (% 1 RM)	Muscle Group Location
Filip-Stachnik et al., 2020 [6]	13	23 ± 0.8	6	45	50	Upper body
Tamilio et al., 2022 [11]	22	20 ± 2	3	45	-	Upper body, lower body
Ferreira et al., 2022 [27]	21	19.6 ± 0.8	6, 8	45	-	Upper body, lower body
Berjisian et al., 2022 [28]	20	24 ± 9	6	60	70	Upper body, lower body
Grgic and Mikulic, 2021 [29]	13	28.5 ± 5	3	60	85	Upper body
Filip-Stachnik et al., 2021 [30]	21	23 ± 0.9	3, 6	60	-	Upper body
Grgic et al., 2020(1) [31]	20	29.3 ± 4.8	3	60	85	Upper body
Grgic et al., 2020(2) [32]	22	AA: 27.0 ± 5.6 AC/CC: 29.8 ± 3.6	3	60	85	Upper body
Grgic et al., 2020(3) [33]	28	25 ± 6	2, 4, 6	60	60	Upper body, lower body
Wilk et al., 2019 [34]	16	24.2 ± 4.2	9, 11	60	50	Upper body
Richardson et al., 2016 [35]	9	24 ± 2	5	60	60	Upper body, lower body
Arazi et al., 2016 [36]	10	16.8 ± 1.23	2, 5	60	60	Lower body
Astorino et al., 2011 [37]	14	23.1 ± 1.1	6	60	70, 80	Upper body, lower body
Astorino et al., 2008 [38]	22	23.4 ± 3.6	6	60	60	Upper body, lower body

Abbreviations: y, year; AA, AA genotype at rs762551; AC/CC, AC/CC genotypes at rs762551; BW, body weight.

**Table 2:** Methodological assessment of randomized controlled trials included in the systematic review using the PEDro scale.

Study	A	B	C	D	E	F	G	H	I	J	K	Score
Filip-Stachnik et al., 2020 [6]	Y	1	1	1	1	1	1	0	0	1	1	8/10
Tamilio et al., 2022 [11]	Y	1	1	1	1	1	1	0	0	1	1	8/10
Ferreira et al., 2022 [27]	Y	1	1	1	1	1	0	0	0	1	1	7/10
Berjisian et al., 2022 [28]	Y	1	1	1	1	1	1	0	0	1	1	8/10
Grgic and Mikulic, 2021 [29]	Y	1	1	1	1	1	1	0	0	1	1	8/10
Filip-Stachnik et al., 2021 [30]	Y	1	1	1	1	1	1	0	0	1	1	8/10
Grgic et al., 2020(1) [31]	Y	1	1	1	1	1	1	0	1	1	1	9/10
Grgic et al., 2020(2) [32]	Y	1	1	1	1	1	1	0	1	1	1	9/10
Grgic et al., 2020(3) [33]	Y	1	1	1	1	1	1	0	1	1	1	9/10
Wilk et al., 2019 [34]	Y	1	1	1	1	1	1	0	0	1	1	8/10
Richardson et al., 2016 [35]	Y	1	1	1	1	1	0	0	0	1	1	7/10
Arazi et al., 2016 [36]	Y	1	1	1	1	1	1	0	0	1	1	8/10
Astorino et al., 2011 [37]	Y	1	1	1	1	1	0	0	0	1	1	7/10
Astorino et al., 2008 [38]	Y	1	1	1	1	1	0	0	0	1	1	7/10

Abbreviations: A, eligibility criteria; B, random allocation; C, concealed allocation; D, baseline comparability; E, blind subjects; F, blind therapists; G, blind assessors; H, adequate follow-up; I, intention-to-treat analysis; J, between-group comparisons; K, point estimates and variability. The total score represents the score of the PEDro scale. Item 1 was not scored. Y: yes.

**Table 3:** Results of Egger’s test (muscle strength).

Std_EFF	Coef.	Std.Err.	t	*p* > |t|	95% CI
Slope	8.410969	0.928141	9.06	0.000	6.461017, 10.36092
Bias	−0.8189293	0.3990989	−2.05	0.055	−1.657405, 0.0195464

Abbreviations: Coef., coefficient; Std.Err., standard error; t, *t*-test statistic; *p*, probability; 95% CI, 95% confidence interval.

**Table 4:** Results of Egger’s test (muscle endurance).

Std_EFF	Coef.	Std.Err.	t	*p* > |t|	95% CI
Slope	1.115718	0.5029182	2.22	0.034	0.0886218, 2.142814
Bias	0.2502451	0.4435465	0.56	0.577	−0.6555977, 1.156088

Abbreviations: Coef., coefficient; Std.Err., standard error; t, *t*-test statistic; *p*, probability; 95% CI, 95% confidence interval.

## Data Availability

All data generated or analyzed during this study are included in the article/Supplementary Material.

## Supplementary Materials

The following supporting information can be downloaded at https://www.mdpi.com/article/10.3390/nu16081146/s1, Figure S1: Meta-regression analysis results of muscle strength; Figure S2: Meta-regression analysis results of muscle endurance; Figure S3: Cochrane risk of bias tool; Figure S4: Funnel plot; Figure S5: Sensitivity analysis results of muscle strength; Figure S6: Sensitivity analysis results of muscle endurance.
