# Supplementary figures and images for: Effects of Acute Ingestion of Caffeine Capsules on Muscle Strength and Muscle Endurance: A Systematic Review and Meta-Analysis
Source: Nutrients. 2024 Apr 12;16(8):1146. doi: 10.3390/nu16081146 (PMC11054210; doi:10.3390/nu16081146)

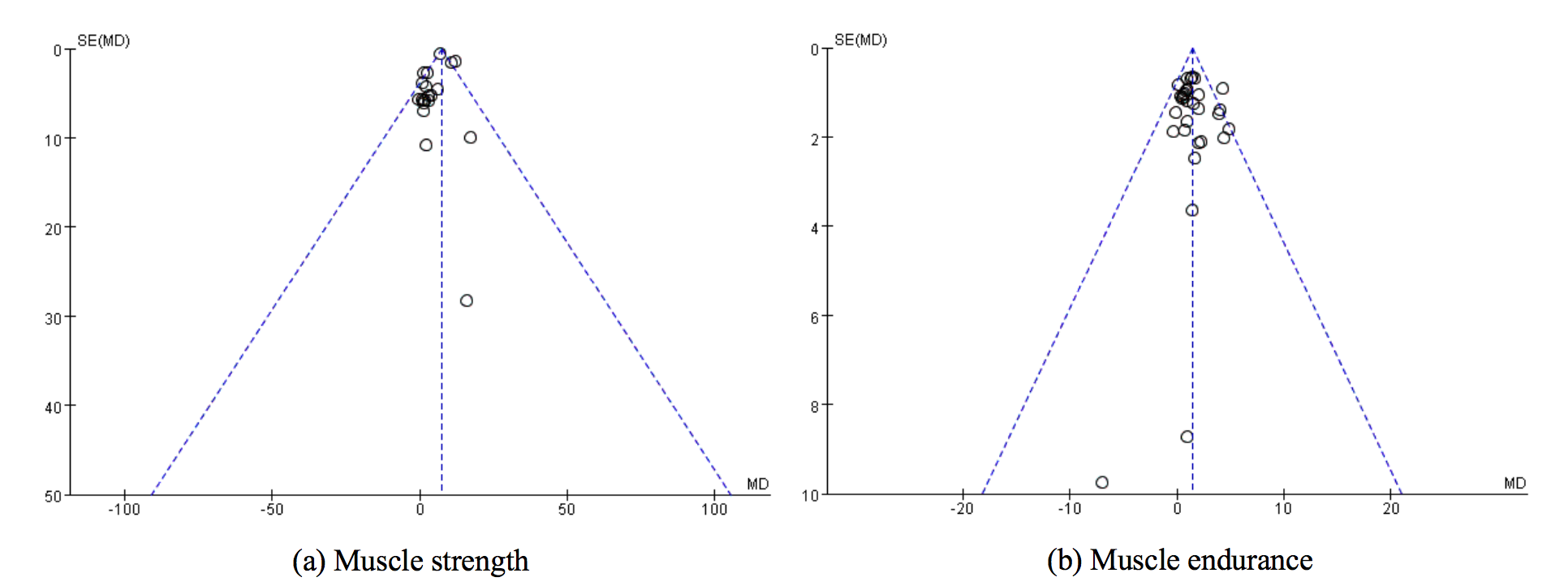

Supplement: Supplementary file 1 [file nutrients-16-01146-s001.zip › Supplementary Material/Figure S4 Funnel plot.tif]

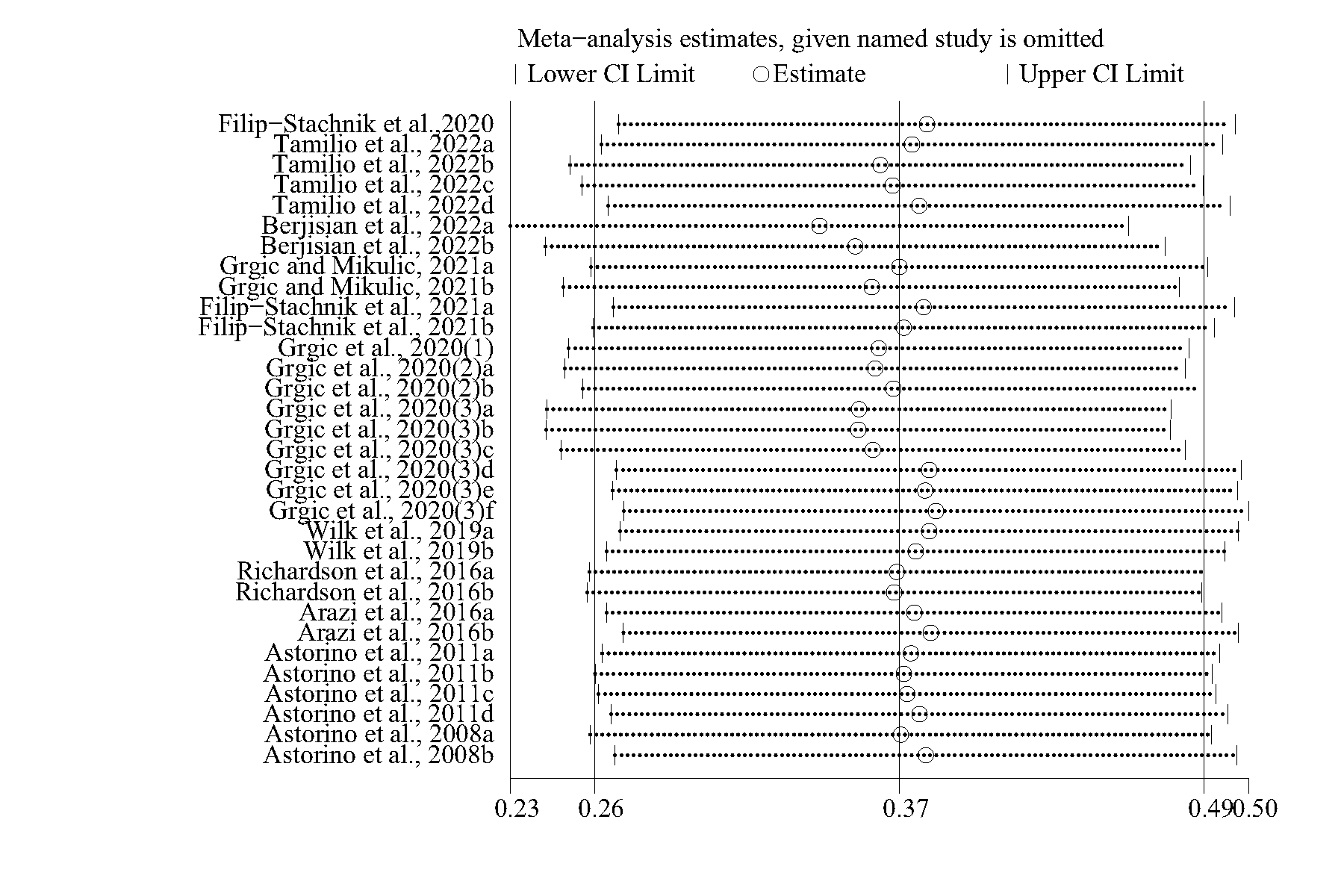

Supplement: Supplementary file 1 [file nutrients-16-01146-s001.zip › Supplementary Material/Figure S6 Sensitivity analysis results of muscle endurance.tif]

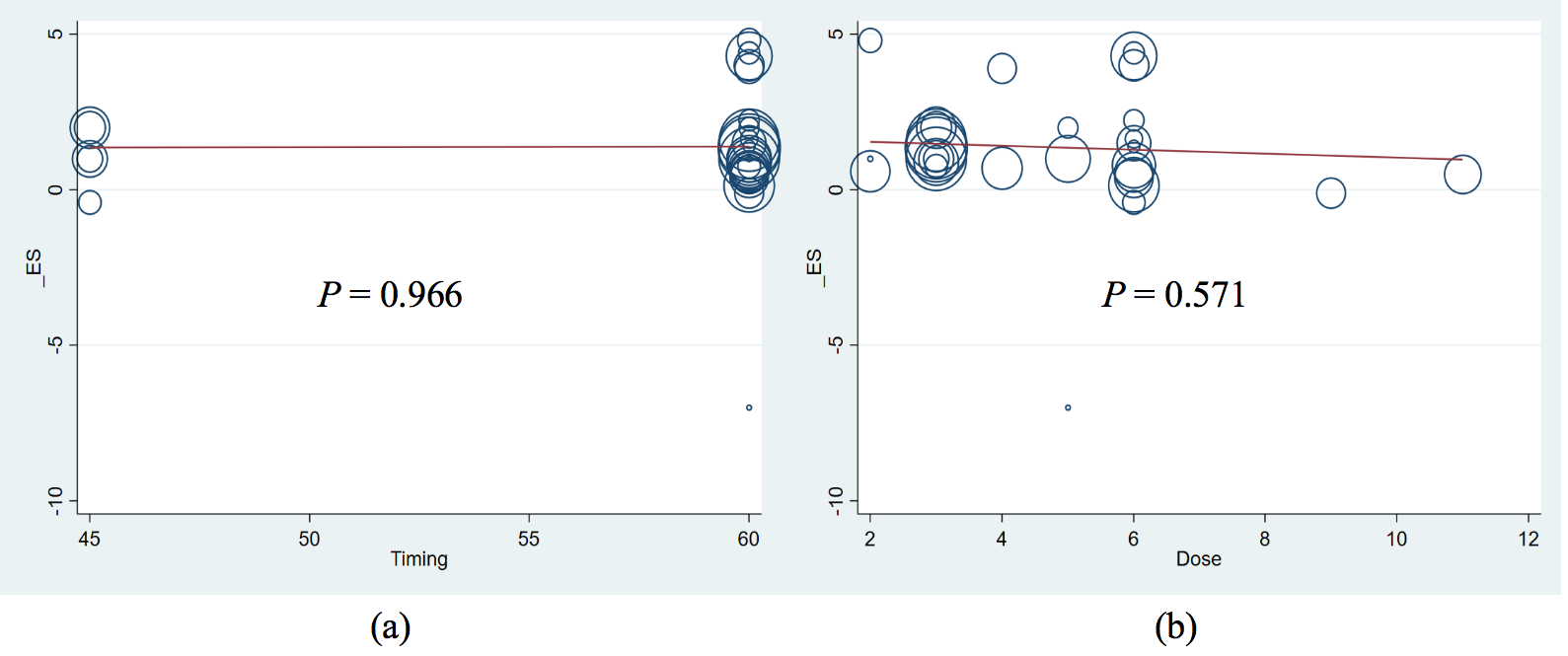

Supplement: Supplementary file 1 [file nutrients-16-01146-s001.zip › Supplementary Material/Figure S2 Meta-regression analysis results of muscle endurance.tif]

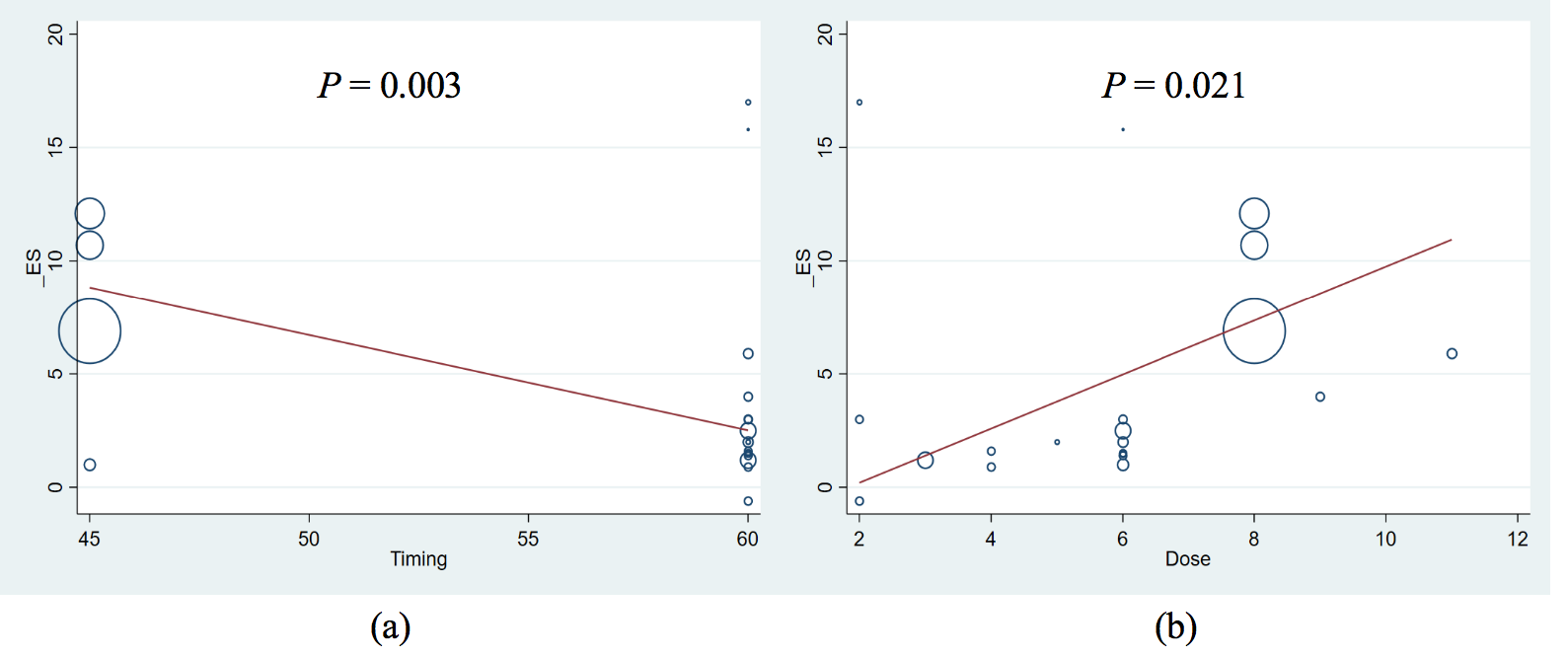

Supplement: Supplementary file 1 [file nutrients-16-01146-s001.zip › Supplementary Material/Figure S1 Meta-regression analysis results of muscle strength.tif]

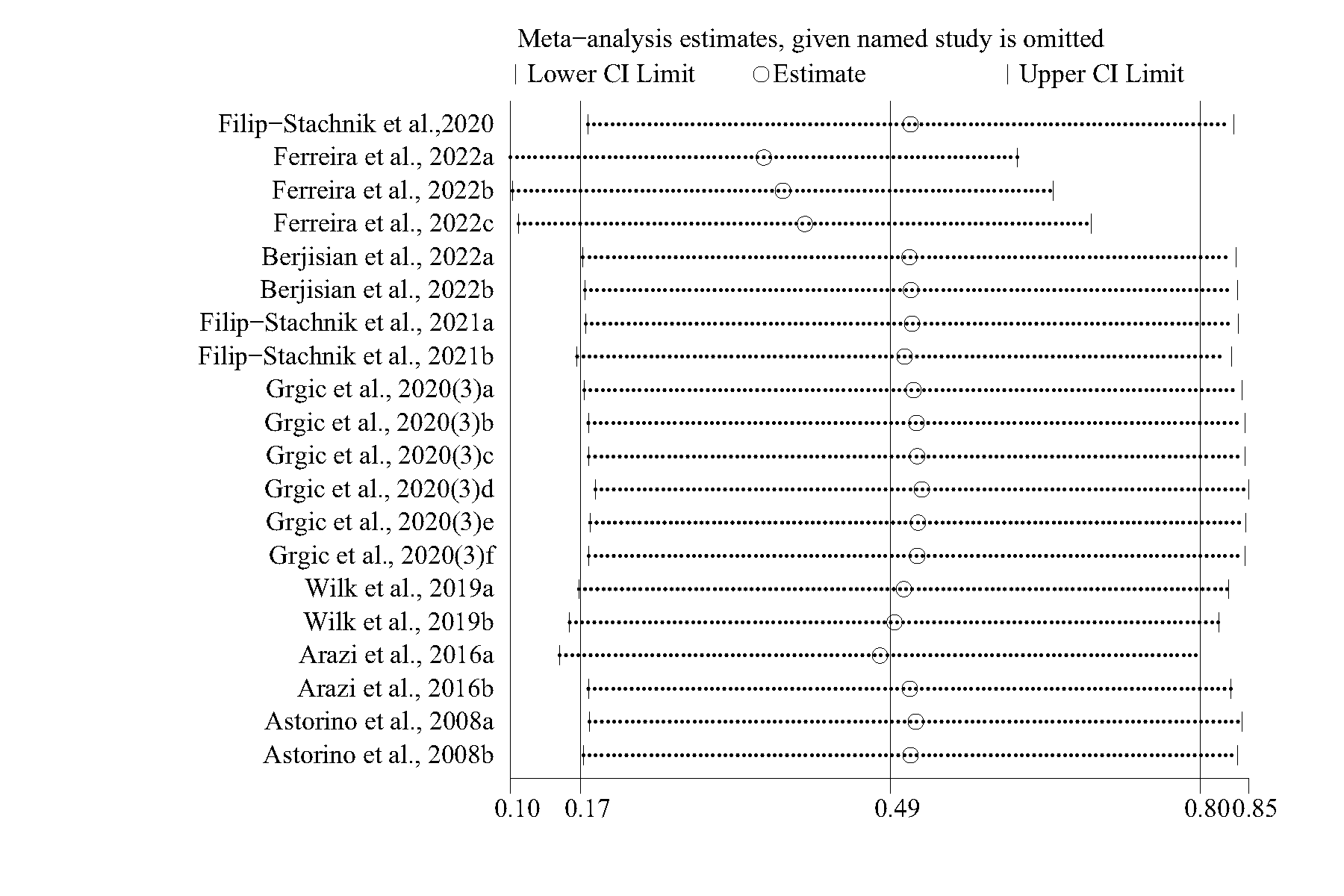

Supplement: Supplementary file 1 [file nutrients-16-01146-s001.zip › Supplementary Material/Figure S5 Sensitivity analysis results of muscle strength.tif]

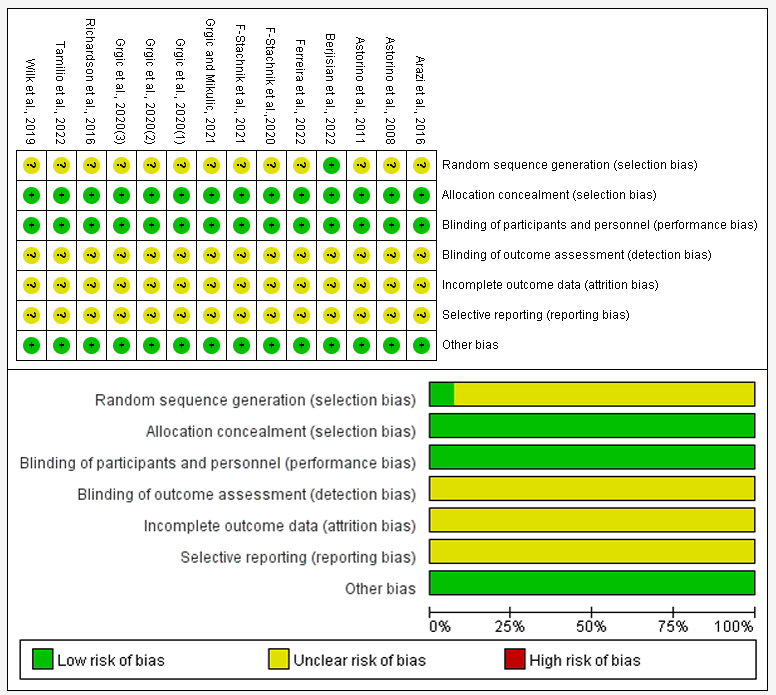

Supplement: Supplementary file 1 [file nutrients-16-01146-s001.zip › Supplementary Material/Figure S3 Results of Cochrane risk of bias tool.tif]
